# Bone marrow‐derived mesenchymal stem cells inhibit NK cell function via Tim‐3/galectin‐9 in multiple myeloma patients

**DOI:** 10.1002/ctm2.1224

**Published:** 2023-03-20

**Authors:** Zhao‐Yun Liu, Nan‐Hao Meng, Pan‐Pan Cao, Yue Jia, Hao Wang, Yun‐He Zhang, Hui Liu, Rong Fu

**Affiliations:** ^1^ Department of Hematology Tianjin Medical University General Hospital, Heping District Tianjin People's Republic of China

Dear Editor,

Our research clarifies that the characteristics of NK cell immunodepleting in the bone marrow micro‐environment of newly diagnosed multiple myeloma (NDMM) patients. Mechanically, bone marrow mesenchymal stem cells (BMSCs) from multiple myeloma (MM) patients can promote NK cell exhaustion via Tim‐3/galectin‐9 through direct or indirect cell‐to‐cell communication.

MM is known as haematological neoplasia, in which aberrant plasma cells develop clonally in the bone marrow, leading to anaemia, renal damage, osteolytic destruction and abnormal immune function.^1^ NK cell is a lymphocyte that kills tumour cells without prior sensitization. The exhaustion of NK cells plays a vital role in MM bone marrow inhibiting the immune environment.^2^ T‐cell immunoglobulin mucin 3 (Tim‐3), which can bind galectin‐9 on myeloma cells, is known to be a negative regulatory molecule of NK cells.^3^ BMSC is crucial for modulating functions of immune cells. Studies have shown that MM‐derived BMSCs can interact with immune cells in the bone marrow micro‐environment through direct or indirect cell‐to‐cell communication, regulating immune cell function and thus influencing the onset and development of MM.^4^, ^5^ However, there is little known about Tim‐3 ligands on BMSCs and the ways interact with NK cells.

First, we used clinical samples to evaluate the NK cell status in MM (Supplemental Table S1). In terms of quantity, the ratios of total NK cells (CD3^−^CD56^+^) and CD56^dim^NK cells (CD16^+^CD56^dim^) were significantly higher in NDMM and complete remission multiple myeloma (CR) than in Healthy donors (HDs), but the ratios of CD56^bright^NK cells (CD16^−^CD56^bright^) were not significantly different between three groups (Supplemental Figure S1A and Supplemental Table S2). In terms of functionality, expression of NK cell functional molecules (CD107a, NKG2D, INF‐γ, and perforin) was lower in NDMM than in HDs (Supplemental Figure S1B‐E and Supplemental Table S3). In short, we observed that NK cells of MM have an increased quantity but a decreased function.

So, why did NK cells functionally exhaust in MM? Single‐cell RNA sequencing datasets GSE188632 (end‐stage MM) and GSE166902 (healthy donor, HD) were reanalysed.^6^ It showed that HAVCR2 gene (Tim‐3) expression of NK cells was higher in MM (Figure 1A–C). GSE27838 (NK cell RNA sequencing data from eight HDs and MM) was also reanalysed.^7^ HAVCR2 gene expression was also higher in MM (Figure 1D), but other immune checkpoints were not significantly different (Supplemental Figure S2). Meanwhile, GSE113736 (BMSCs RNA sequencing data) was also reanalysed to verify Tim‐3 ligands expression.^8^ LGALS9 (galectin‐9) expression was higher in MM than in HDs, while other Tim‐3 ligands (HMGB1, CEACAM1, and PtdSer) had no significant differences (Figure 1E). Using the MMRF‐COMMPASS database, we demonstrated that survival was shorter in the high Tim‐3 group than in the low Tim‐3 group (Figure 1F). These suggested that Tim‐3 can influent the early survival of MM and Tim‐3 may regulate NK cell function by interaction with GSE113736 in BMSCs.

**FIGURE 1:**
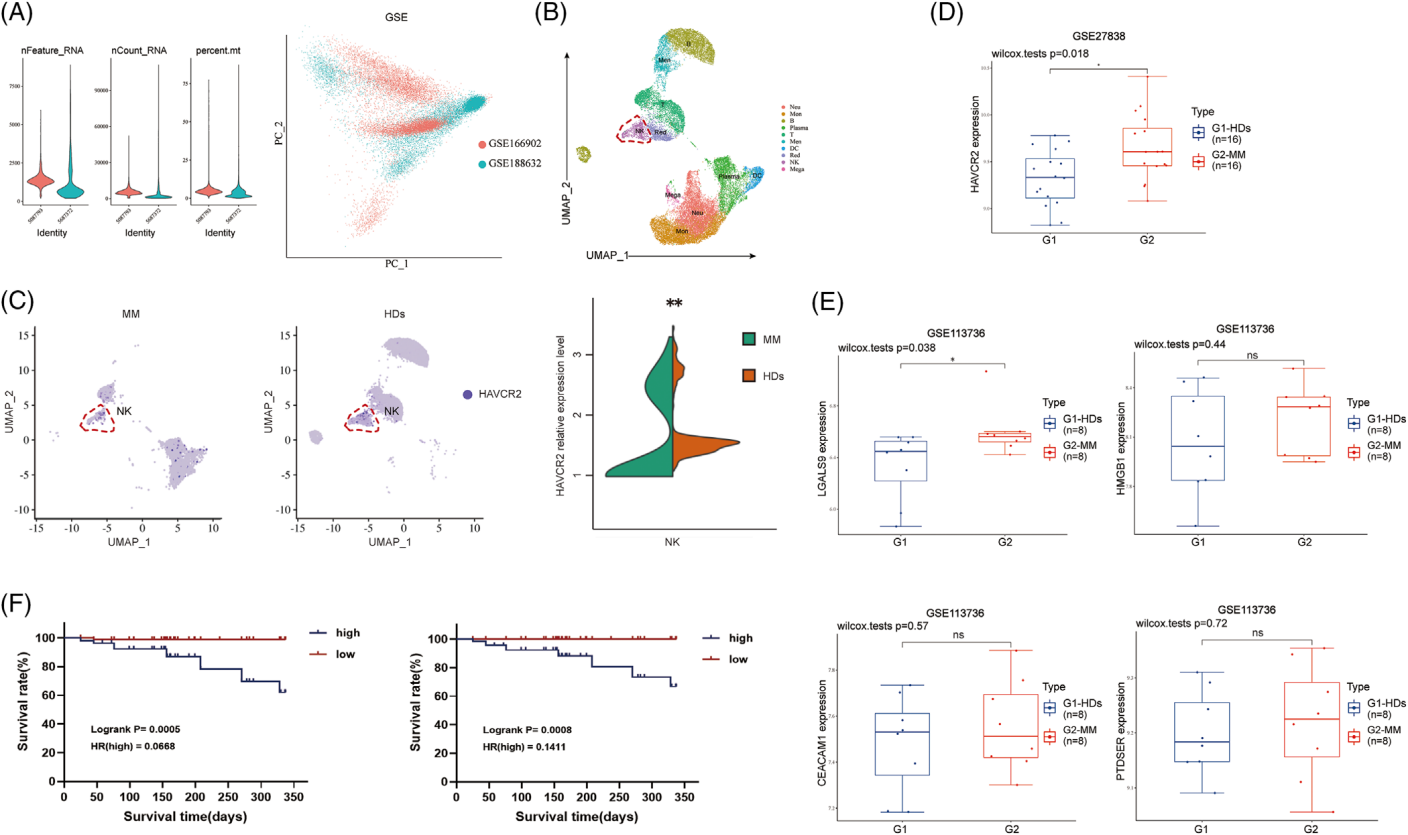
Gene expression level of NK cell HAVCR2 (Tim‐3) in MM patients is increased and predicted bad prognosis. (A) Visual QC metrics and data cleaning: filter the data based on the gene data detected in each cell of GSE188632 (MM) and GSE166902 (HDs), the total number of molecules detected in the cells, and the percentage of mitochondrial genomes in the cells; Batch removal: multiple PC populations with large variance that can be used as anchor points after PCA analysis (gene populations), showing the PCA analysis graph after combining PC1 and PC2 parts to remove the batch effect, different colours represent different batch data; (B) The results of grouping by UMAP, different numbers or colours represent different cell populations, and then according to Top10 marker genes, the artificially annotated cell subpopulation map was realized, different colours represent different cell groups; (C) Showing the expression map of HAVCR2 (Tim‐3) in different subgroups, blue colour represents the expression of the gene, the results show that the expression of HAVCR2 gene is higher in MM patients than HDs; (D) GSE27838 shows that the NK cells of MM patients; (E) GSE113736 showed that LGALS9 (galectin‐9) gene expression was significantly higher in BMSCs of MM patients than in HDs; (F) Analysis of the MMRF‐COMPASS database showed that early survival was shorter in MM patients with high HAVCR2 gene expression.

Next, we used clinical samples to verify the significant high Tim‐3 expression in MM. Tim‐3 expression of total NK and CD56^dim^NK cells was significantly greater in NDMM than in CR and HDs. Tim‐3 expression of CD56^bright^NK cells was also greater in NDMM than in HDs (Supplemental Figure S3A and Supplemental Table S4). As for functions of Tim‐3‐positive NK cells (CD3^−^CD56^+^Tim‐3^+^), the expression of CD107a, NKG2D and INF‐γ on Tim‐3‐positive NK cells considerably decreased in NDMM than HDs (Supplemental Figure S3B and Supplemental Table S5). Then, functions of Tim‐3‐positive NK and Tim‐3‐negative NK cells in each MM patient were compared. Expression of NK cell functional molecules on Tim‐3‐positive NK cells significantly decreased than those on Tim‐3‐negative NK cells (Supplemental Figure S3C). Tim‐3 expression and NK cell functional molecules expression were all negatively correlated (Supplemental Figure S3D). These results confirmed that Tim‐3 was a negative regulatory molecule for NK cells in MM.

Next, Tim‐3 ligands on BMSCs and human MM cell lines (U266, RPMI‐8226) were detected via flow cytometry. Galectin‐9 was highly expressed on BMSCs compared to HMGB1, CEACAM1 and PtdSer. However, no significant differences were seen in MM cells (Figure 2A; Supplemental Figure S4 and Supplemental Table S6). Furthermore, we observed the expression of galectin‐9 on BMSCs was positively correlated with M protein and bone marrow plasma cell ratio in MM patients (Figure 2B; Supplemental Figures S7 and S8). We speculated that BMSCs regulate NK cells via Tim‐3/ galectin‐9 and confirmed it by using in vitro co‐culture. The BMSCs/NK co‐culture systems were constructed (Figure 2C). Expression of CD107a, NKG2D, INF‐γ and perforin on NK cells was significantly decreased after co‐culture with BMSCs for 6 days, while these markers were restored in the Tim‐3 inhibitor group (Figure 2D and Supplemental Table S7). U266 cells were added to each co‐culture group, and the apoptosis of U266 cells was examined after 72 h. The apoptosis ratios of U266 were significantly decreased after co‐culture with BMSCs and restored in the Tim‐3 inhibitor group (Figure 2E). Moreover, the NK cells functional molecules expression and the galectin‐9 expression on BMSCs were negatively correlated (Figure 2F). Those results indicated that BMSCs can negatively regulate NK cell function by Tim‐3/galectin‐9.

**FIGURE 2:**
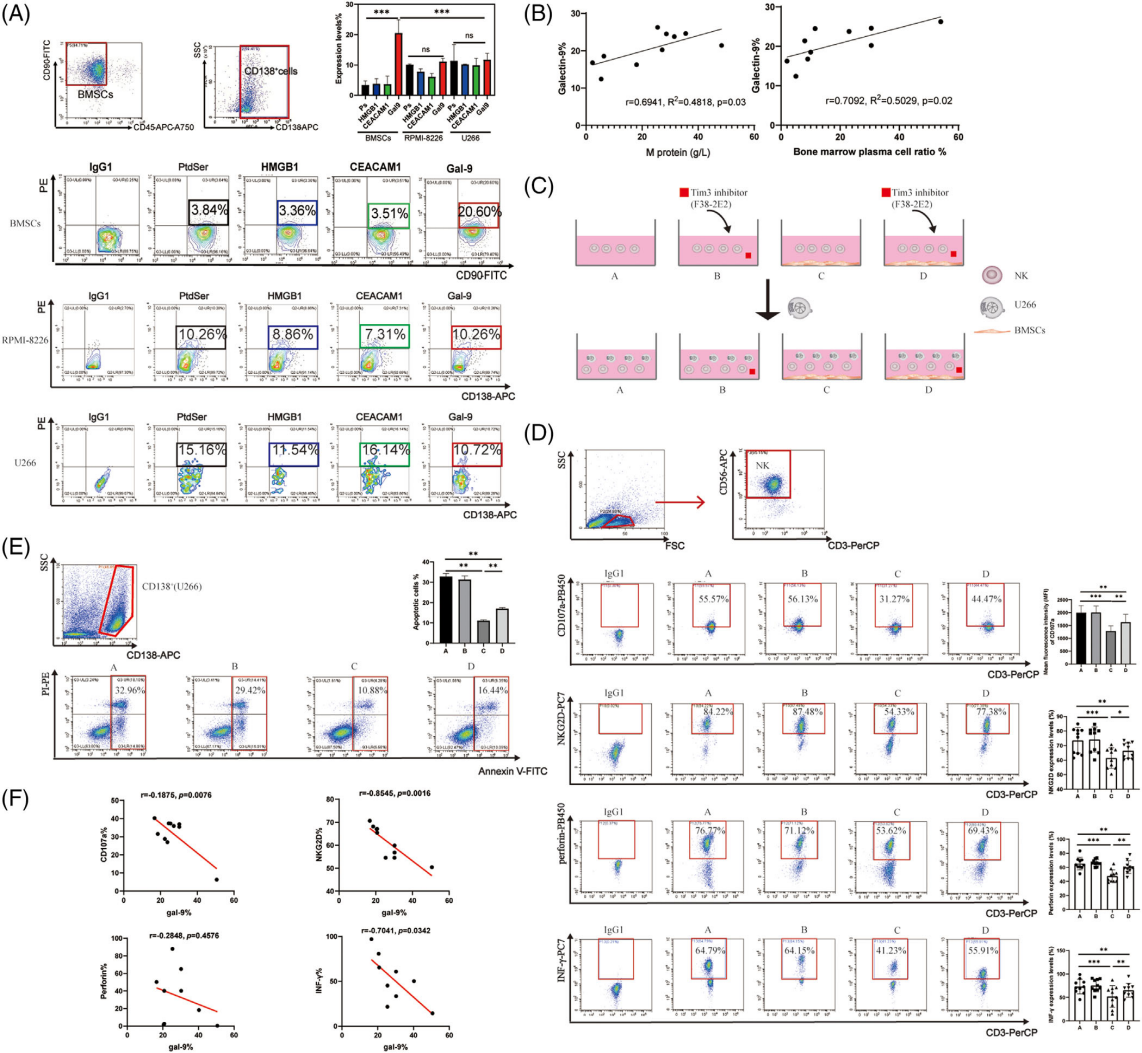
BMSCs regulated NK‐92 cells function by Tim‐3/galectin‐9 in a direct co‐culture system. (A) Expression of Tim‐3 ligands protein on the surface of BMSCs and MM cell line U266, RPMI‐8226 cells, the Tim‐3 ligands include galectin‐9, HMGB1, CEACAM‐1, and PtdSer; (B) Expression of galectin‐9 on BMSCs increased with the increase of MM burden; (C) The grouping methods of BMSCs/NK co‐culture systems; (D) FCM analysis CD107a, NKG2D, INF‐γ and perforin expression on NK cells after direct co‐culture of BMSCs and NK cells, functional molecules expressions of NK cells were reduced after co‐culture and restored after blocking Tim‐3 (*n* = 10); (E) The tumour‐killing ability of NK cells was diminished after co‐culture and partially restored after blocking Tim‐3 (*n* = 3); (F) Expression of functional molecules on NK cells and expression of galectin‐9 on BMSCs in the co‐culture system were negatively correlated.

Whether BMSCs only regulated NK cells through direct contact? The BMSCs/NK indirect co‐culture systems were constructed (Figure 3A). Expression of CD107a, NKG2D, INF‐γ, and perforin was significantly decreased after co‐culture with BMSCs, and these markers were restored in the Tim‐3 inhibitor group and exosome inhibitor group (Figure 3B and Supplemental Table S8). The apoptosis of U266 was significantly decreased after co‐culture with BMSCs and restored in the Tim‐3 inhibitor group and exosome inhibitor group (Figure 3C). Then, we knocked down the galectin‐9 on BMSCs to purified BMSCs‐derived exosomes (Figure 3D; Supplemental Figures S5 and S6A; Supplemental Tables S9 and S10), Western‐blot showed galectin‐9 expression on galectin‐9 knockdown BMSCs‐derived exosomes was decreased significantly than BMSCs‐derived exosomes^9^, ^10^ (Supplemental Figure S6B). After that, NK cells were co‐cultured with BMSCs, galectin‐9 knockdown BMSCs, BMSCs‐derived exosomes, and galectin‐9 knockdown BMSCs‐derived exosomes respectively (Figure 3E). Expression of NK cell functional molecules was significantly restored in the galectin‐9 knockdown BMSCs group compared to the normal BMSCs group; furthermore, expressions of NK cell functional molecules were significantly decreased after co‐culture with BMSCs‐derived exosomes, and these markers were restored in the galectin‐9 knockdown BMSCs‐derived exosomes group (Figure 3F). The apoptosis of U266 was significantly increased in the galectin‐9 knockdown BMSCs‐derived exosomes group than co‐cultured with BMSCs‐derived exosomes (Figure 3G). Finally, we detected the related protein changes in the Tim‐3/galectin‐9 pathway, Notch1 and EOMES expression decreased when NK co‐culture with BMSCs, which indicated that the Tim‐3/galectin‐9 pathway might exhaust NK cells through down‐regulating Notch1/EOMES (Figure 3H).

**FIGURE 3:**
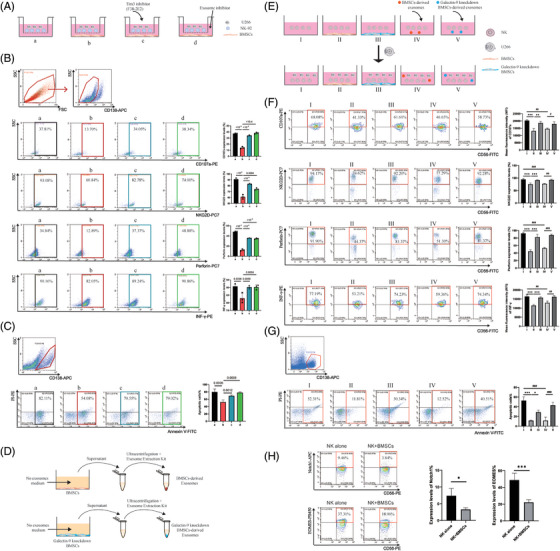
BMSCs regulated NK‐92 cells function by the Tim‐3/galectin‐9 pathway. (A) Grouping of BMSCs and NK cells in indirect co‐culture, after the third‐generation BMSCs were laid flat in the lower layer of the well plates (groups b–d) until the BMSCs had grown to maturity against the wall, the medium was removed and replaced with exosome‐free medium, and equal proportions of NK cells and U266 cells were added to the upper chamber (a–d), respectively, F38‐2E2 was added to the upper chamber in group c, and GW4869 (exosome inhibitor) was added to the lower layer of the well plate in group d. The culture was continued for 3 days for subsequent experiments; (B) In the absence of direct contact between BMSCs and NK cells, BMSCs can still inhibit NK functional molecule expression, and blocking Tim‐3 or BMSCs‐derived exosomes can counteract some of the inhibition (*n* = 3); (C) Tumour‐killing ability of NK cells in indirect co‐culture (*n* = 3); (D) BMSCs‐derived exosomes were isolated by ultracentrifugation and exosome extraction kit; (E) Grouping of BMSCs‐derived exosomes and NK cells, I: NK alone, II: NK+BMSCs, III: NK+galectin‐9 knockdown BMSCs, IV: NK+BMSCs‐derived exosomes, V: NK+galectin‐9 knockdown BMSCs‐derived exosomes; (F) The inhibitory effect of galectin‐9 knockdown BMSCs on NK cell function was weaker than that of normal BMSCs, BMSCs‐derived exosomes can also inhibit the NK function and the inhibitory effect of galectin‐9 knockdown BMSCs‐exosomes was weaker (*n* = 3); (G) Tumour‐killing ability of NK cells in BMSCs‐derived exosomes/NK coculture system (*n* = 3); (H) Notch1 and EOMES expression decreased when NK co‐culture with BMSCs.

In summary, NK cells are exhausted in the bone marrow micro‐environment of MM patients. Mechanically, MM‐derived BMSCs can inhibit the immune response of NK cells via Tim‐3/galectin‐9, both through direct cell‐to‐cell contact and indirect contact mediated by exosomes, and the application of Tim‐3 inhibitors or exosome inhibitors can restore the exhaustion of NK cells (Figure 4).

**FIGURE 4:**
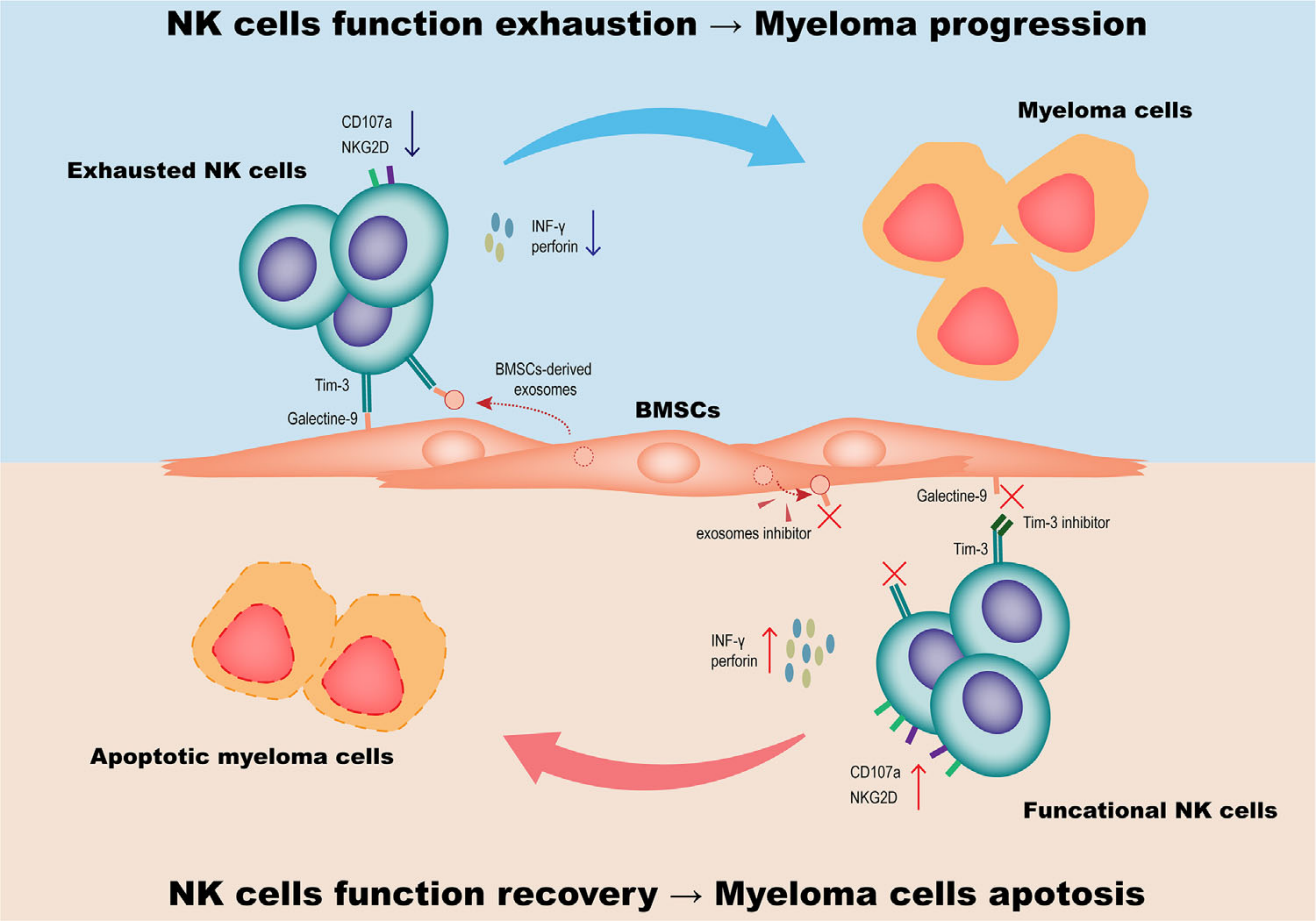
Blocking the binding of Tim‐3 of NK cells and galectin‐9 of BMSCs or blocking the BMSCs‐derived exosomes can enhance the function of NK cells, resulting in increased apoptosis of myeloma cells.

## CONFLICT OF INTEREST STATEMENT

The authors declare no conflicts interests.

## Supporting information

Supporting information

Supporting information

Supporting information
