# Supplementary material for: Bone marrow‐derived mesenchymal stem cells inhibit NK cell function via Tim‐3/galectin‐9 in multiple myeloma patients
Source: Clin Transl Med. 2023 Mar 20;13(3):e1224. doi: 10.1002/ctm2.1224 (PMC10026087; doi:10.1002/ctm2.1224)
Supplement: Supplementary file 1 — Supporting information [file CTM2-13-e1224-s003.docx]

**Supplemental Figures**


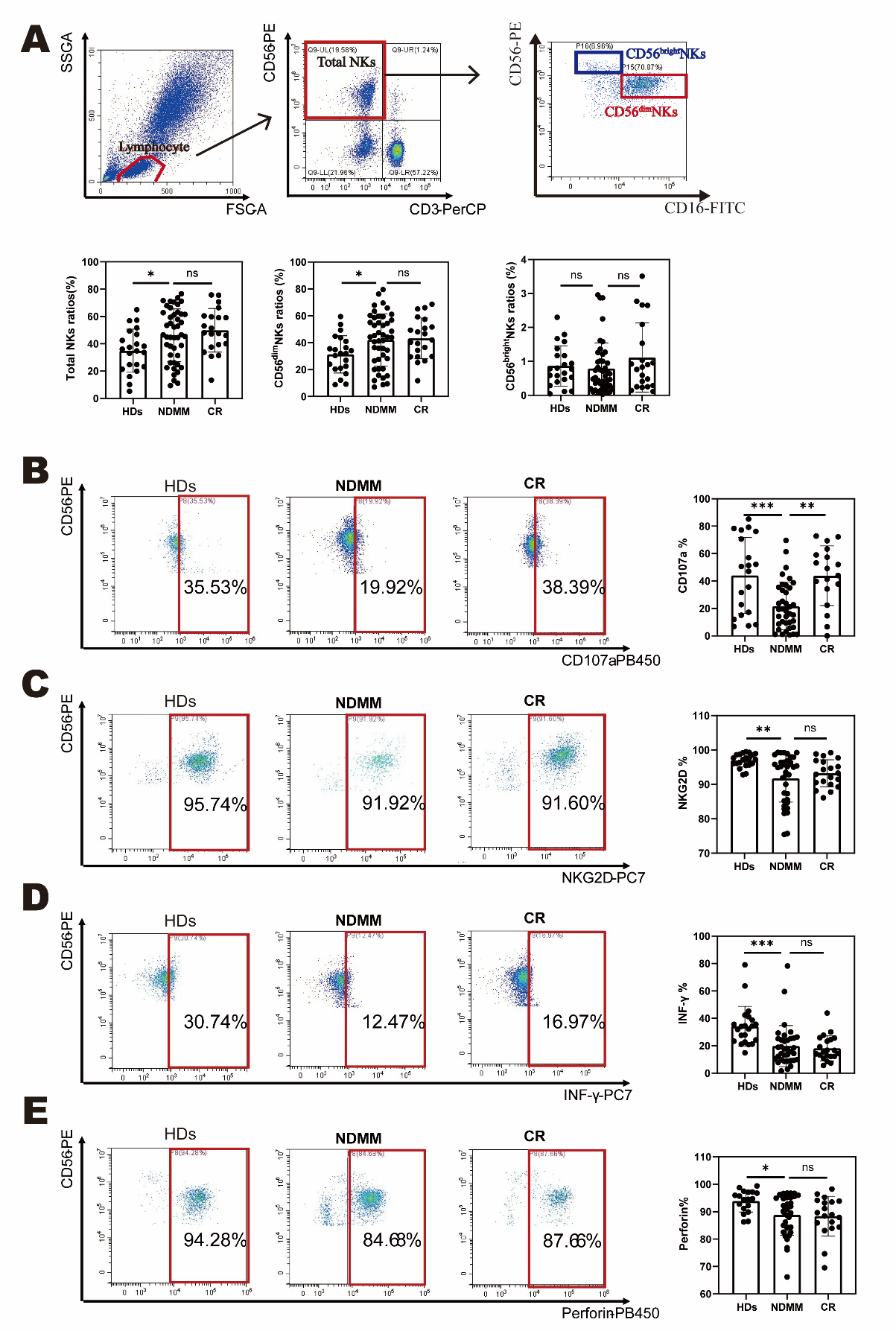


**Figure S1.** Quantity and function of bone marrow NK cells in MM patients. A, FCM analysis the quantities of total NK cells, CD56^dim^NK cells and CD56^bright^NK cells; B, C, and D, FCM analysis functions of bone marrow NK cells in MM patients, NK cell function as demonstrated by the expression of CD107a, NKG2D, INF-γ, and Perforin.


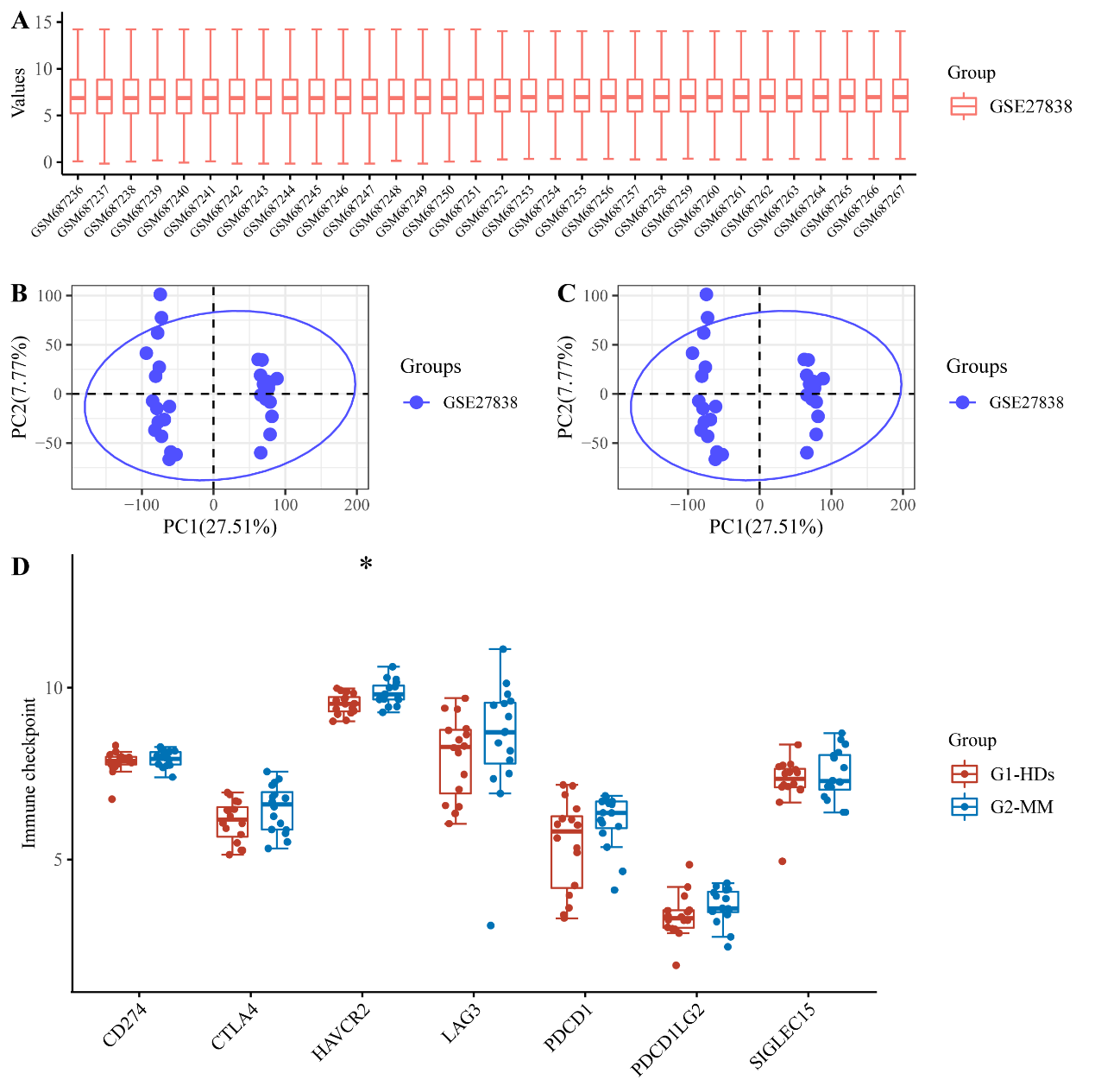


**Figure S2.** Analysis of GSE27838 data showed that significantly higher RNA expression of HAVCR2 (Tim-3) in a variety of immunodetectable sites in NK cells from MM patients than from healthy controls (HDs).


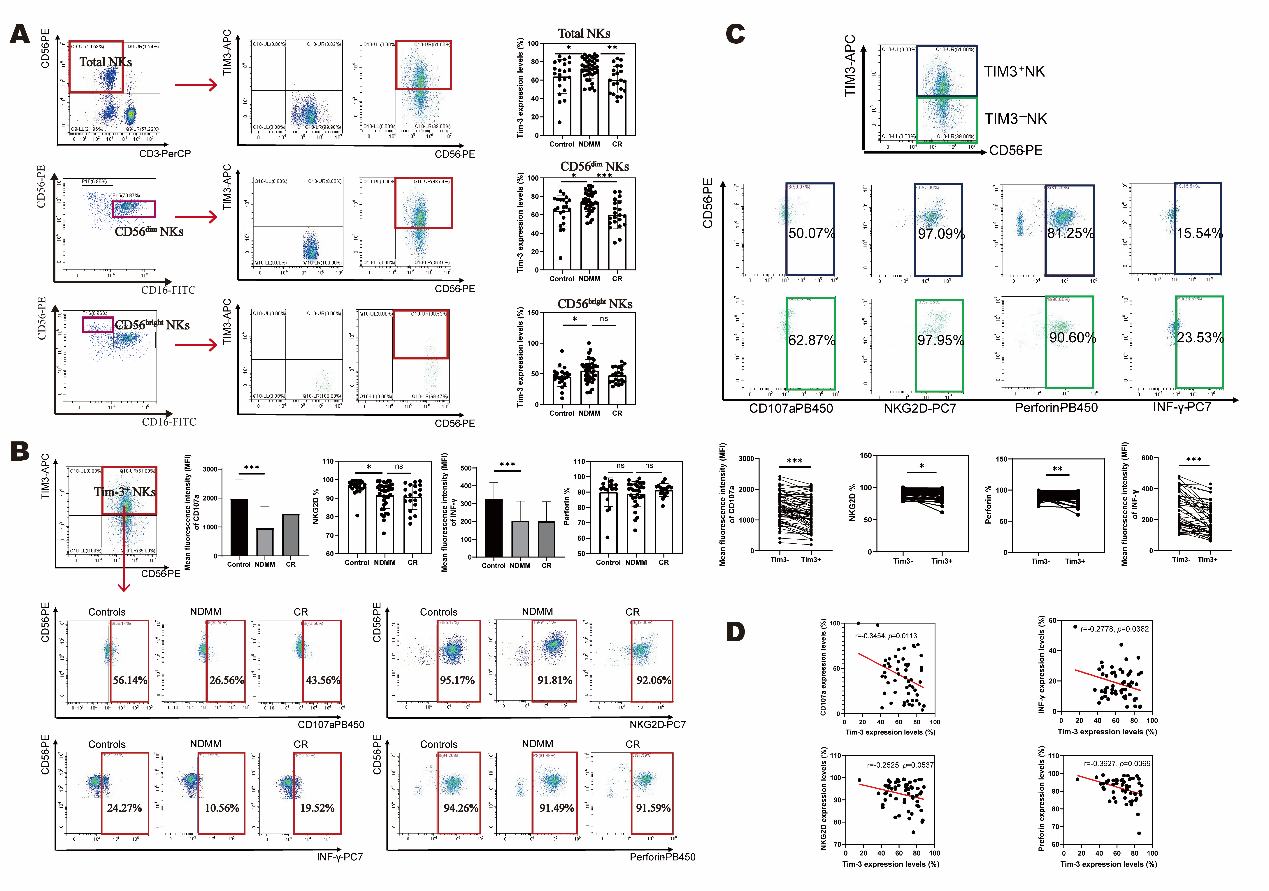


**Figure S3.** Tim-3 involved in the exhaust of NK cell from MM patients. A, Expression of Tim-3 on total NK cells, CD56dimNK cells and CD56brightNK cells; B, Expression of CD107a, NKG2D, INF-γ, and perforin on Tim-3-positive NK cells; C, Paired comparison of CD107a, NKG2D, INF-γ, and perforin expression in Tim-3-positive NK cells and Tim-3-negative NK cells; D, Correlation between Tim-3 expression in NK cells and NK cell functions.


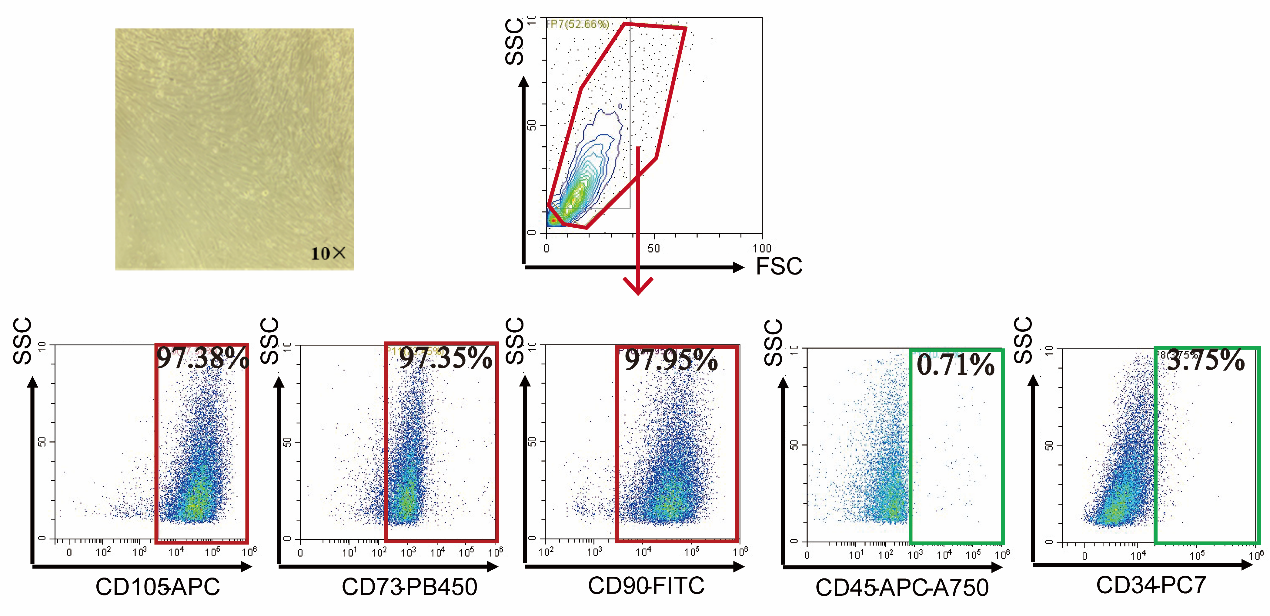


**Figure S4.** Observation of BMSCs morphology under inverted microscope. BMSCs cells were homogeneous and uniformly long spindle-shaped. FCM was used to identify surface-specific antibodies of BMSCs and verify the purity. The results showed that BMSCs highly expressed CD73, CD90, CD105 and did not express CD34 and CD45, and the purity of BMSCs induced in this study was >95%.


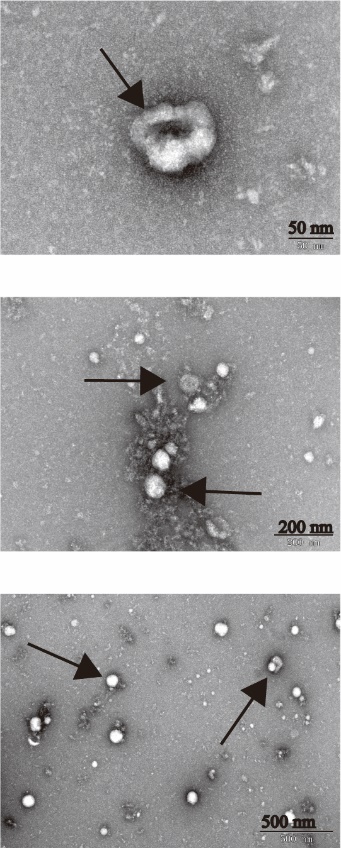


**Figure S5**

TEM images of BMSCs-derived exosomes


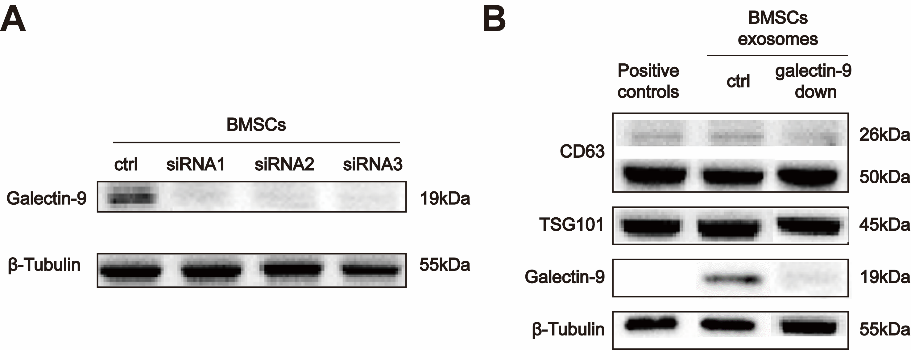


**Figure S6.** A, Galectin-9 protein was knockdown in BMSCs via siRNA; B, Identifying BMSCs-derived exosomes with standard positive controls, then detecting galectin-9 protein expression in BMSCs-derived exosomes and galectin-9 knockdown BMSCs- derived exosomes.


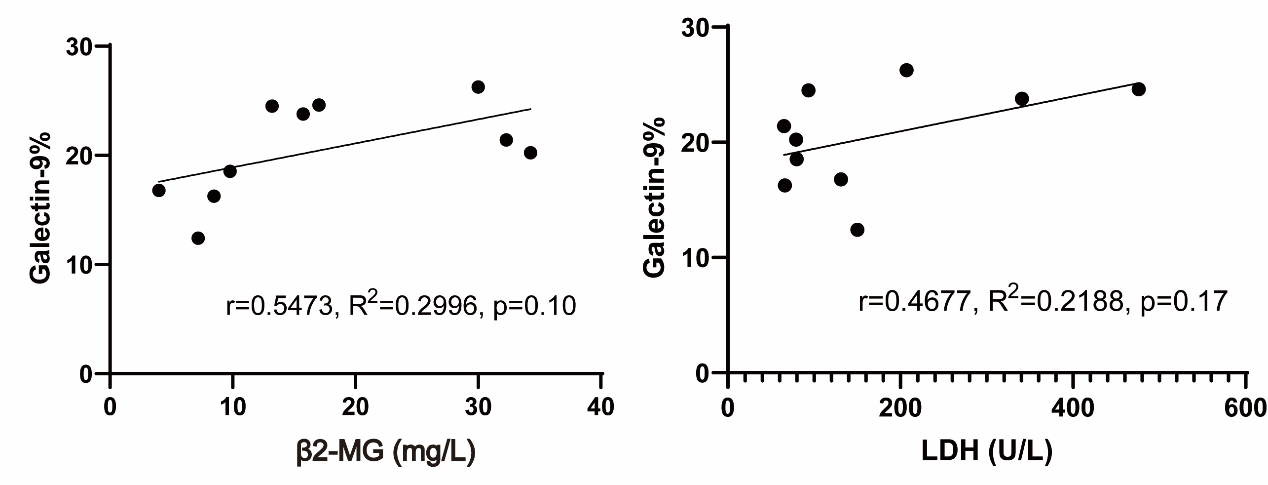


**Figure S7**

Correlation of galectin-9 expression on MM-derived BMSCs and β2-MG levels and LDH levels in MM patients.


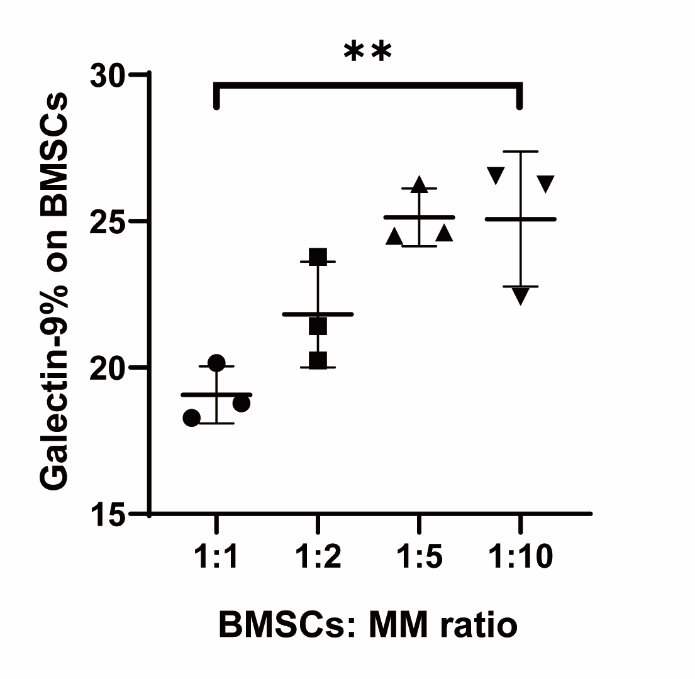


**Figure S8**

Expression of Galectin-9 on BMSCs were increased with increasing MM concentration in co-culture system.
