# Supplementary material for: Bone marrow‐derived mesenchymal stem cells inhibit NK cell function via Tim‐3/galectin‐9 in multiple myeloma patients
Source: Clin Transl Med. 2023 Mar 20;13(3):e1224. doi: 10.1002/ctm2.1224 (PMC10026087; doi:10.1002/ctm2.1224)
Supplement: Supplementary file 2 — Supporting information [file CTM2-13-e1224-s001.docx]

**Supplemental Tables**

**Table S1. The characters of 45 NDMM patients.**

|  | **N** | **%** |
| --- | --- | --- |
| **Total number** | 45 |  |
| **Gender (M/F)** | 28/17 | 62/38 |
| **Age, median (min, max)** | 62 (32-77) |  |
| **Type** | IgG (25), IgA (12), IgD (1), IgE (1), Light chain (6) | IgG (56), IgA (27), IgD (2), IgE (2), Light chain (13) |
| **D-S stage** |  |  |
| **I** | 8 | 18 |
| **II** | 10 | 22 |
| **III** | 27 | 60 |
| **R-ISS stage** |  |  |
| **I** | 5 | 11 |
| **II** | 12 | 27 |
| **III** | 28 | 62 |

**Table S2. NK cells quantities in MM.**

| **Group** | **NK cells quantities, %** | | | ***P* value** |
| --- | --- | --- | --- | --- |
|  | **NDMM** | **CR** | **HDs** |  |
| **Total NK** | 46.27±19.29 | 49.85±16.00 | 34.90±15.78 | 0.0152 |
| **CD56dimNK** | 42.00±19.36 | 43.25±15.15 | 31.35±13.88 | 0.0364 |
| **CD56brightNK** | 0.7873±0.7566 | 1.112±1.019 | 0.8645±0.5931 | 0.3045 |

**Table S3. NK cells functions in MM.**

| **Group** | **NK cells functions, %** | | | ***P* value** |
| --- | --- | --- | --- | --- |
|  | **NDMM** | **CR** | **HDs** |  |
| **CD107a** | 21.66±17.39 | 43.86±21.76 | 44.08±27.69 | 0.0001 |
| **NKG2D** | 91.76±6.913 | 93.21±3.939 | 96.97±1.936 | 0.0023 |
| **INF-γ** | 19.79±14.99 | 18.10±9.124 | 34.20±14.59 | 0.0002 |
| **Perforin** | 88.81±7.383 | 88.34±7.258 | 93.89±3.978 | 0.0191 |

**Table S4. Expression levels of Tim-3 on NK cells.**

| **Group** | **Tim-3 expression level, %** | | | ***P* value** |
| --- | --- | --- | --- | --- |
|  | **NDMM** | **CR** | **HDs** |  |
| **Total NK** | 72.61±11.16 | 60.47±14.39 | 63.93±18.38 | 0.0046 |
| **CD56dimNK** | 74.31±10.71 | 60.10±15.28 | 64.48±16.77 | 0.0005 |
| **CD56brightNK** | 55.26±17.89 | 47.66±12.88 | 45.13±15.68 | 0.0448 |

**Table S5. Functions of Tim-3^+^NK cells.**

| **Group** | **CD3^−^CD56^+^Tim-3^+^NK cells functions, %** | | | ***P* value** |
| --- | --- | --- | --- | --- |
|  | **NDMM** | **CR** | **HDs** |  |
| **CD107a** | 24.68±19.40 | 37.52±17.88 | 39.61±25.84 | 0.0259 |
| **NKG2D** | 91.58±7.104 | 90.73±6.844 | 96.20±3.882 | 0.0081 |
| **INF-γ** | 15.28±9.605 | 18.19±10.07 | 26.53±11.17 | 0.0008 |
| **Perforin** | 88.80±8.010 | 91.31±4.241 | 89.95±9.277 | 0.5589 |

**Table S6. Expression levels of Tim-3 ligands.**

| **Group** | **Tim-3 ligands expression levels, %** | | | | ***P* value** |
| --- | --- | --- | --- | --- | --- |
|  | **Galectin-9** | **HMGB1** | **CEACAM1** | **PtdSer** |  |
| **BMSCs** | 20.48±4.458 | 3.771±1.752 | 3.717±2.622 | 3.364±1.482 | <0.0001 |
| **U266** | 11.11±1.121 | 7.797±0.9293 | 6.053±1.139 | 10.10±0.2048 | 0.1423 |
| **RPMI-8266** | 11.75±2.093 | 10.19±0.0707 | 9.880±2.348 | 11.35±5.388 | 0.9140 |

**Table S7. NK cell functions in direct co-culture of BMSCs/NK.**

| **Group** | **A** | **B** | **C** | **D** | ***P* value** |
| --- | --- | --- | --- | --- | --- |
| **CD107a%** | 55.46±7.676 | 55.60±7.162 | 35.66±5.593 | 45.09±8.530 | <0.0001 |
| **NKG2D%** | 73.56±8.834 | 74.20±8.664 | 61.46±7.065 | 66.42±5.445 | <0.0001 |
| **INF-γ%** | 73.15±15.59 | 75.16±13.32 | 51.79±23.19 | 65.21±14.84 | <0.0001 |
| **Perforin%** | 65.33±8.754 | 66.31±4.536 | 47.58±8.895 | 60.24±9.036 | <0.0001 |

**Table S8. NK cell functions in indirect co-culture of BMSCs/NK.**

| **Group** | **a** | **b** | **c** | **d** | ***P* value** |
| --- | --- | --- | --- | --- | --- |
| **CD107a%** | 37.28±0.7537 | 14.07±1.651 | 34.09±1.061 | 38.06±1.515 | <0.0001 |
| **NKG2D%** | 90.93±1.905 | 61.16±3.740 | 83.27±0.7778 | 74.17±2.006 | <0.0001 |
| **INF-γ%** | 90.79±1.427 | 82.97±3.274 | 90.23±1.027 | 90.25±1.599 | 0.0041 |
| **Perforin%** | 48.25±1.223 | 12.18±1.940 | 35.76±0.4924 | 48.87±1.960 | <0.0001 |

**Table S9. Antibodies and reagents.**

| **FCM antibodies** | | |
| --- | --- | --- |
| CD3 | PerCP | BD, USA |
| CD56 | PE | BD, USA |
| CD16 | FITC | BD, USA |
| Tim-3 | APC | BD, USA |
| Galectine-9 | PE | Miltenyi Biotec, German |
| HMGB-1 | PE | Miltenyi Biotec, German |
| CEACAM-1 | PE | Miltenyi Biotec, German |
| PtdSer | PE | Miltenyi Biotec, German |
| CD107a | PB450 | BD, USA |
|  | PE | BD, USA |
| NKG2D | PC7 | BD, USA |
| INF-γ | PC7 | BD, USA |
|  | PE | BD, USA |
| Perforin | PB450 | BD, USA |
|  | PC7 | BD, USA |
| CD34 | PC7 | BD, USA |
| CD45 | APC-CY7 | BD, USA |
| CD90 | FITC | BD, USA |
| CD105 | APC | BD, USA |
| CD73 | PB450 | BD, USA |
| CD138 | APC | Miltenyi Biotec, German |
| **Tim-3 inhibitor** | | |
| F38-2E2 | BioLegend, USA | |
| **Exosome inhibitor** | | |
| GW4869 | Umibio, China | |
| **Western-blot antibodies** | | |
| CD63 | Umibio, China | |
| TSG101 | Umibio, China | |
| Galectine-9 | Abcam, USA | |
| β-Tubulin | Cell Signaling Technology, USA | |

**Table S10. Sequence of LGALS9 (Galectin-9).**

| Gene Name | Sequence | |
| --- | --- | --- |
|  | Sense(5’-3’) | Antisense(5’-3’) |
| LGALS9-homo-246 | CCAGGUUUGCUGUGAACUUTT | AAGUUCACAGCAAACCUGGTT |
| LGALS9-homo-378 | GGAAGACACACAUGCCUUUTT | AAAGGCAUGUGUGUCUUCCTT |
| LGALS9-homo-954 | GCAACACCCAGAUCGACAATT | UUGUCGAUCUGGGUGUUGCTT |
