# Supplementary material for: Bone marrow‐derived mesenchymal stem cells inhibit NK cell function via Tim‐3/galectin‐9 in multiple myeloma patients
Source: Clin Transl Med. 2023 Mar 20;13(3):e1224. doi: 10.1002/ctm2.1224 (PMC10026087; doi:10.1002/ctm2.1224)
Supplement: Supplementary file 3 — Supporting information [file CTM2-13-e1224-s002.docx]

**MATERIALS AND METHODS**

**1 Patients**

The subjects of this study were taken from 66 patients with MM who attended the Department of Hematology, General Hospital of Tianjin Medical University from September 2020 to December 2021, including 45 patients with newly diagnosed multiple myeloma (NDMM) and 21 patients who achieved complete remission (CR), and 22 age- and sex-matched people were selected as healthy controls (HDS). The clinical information of all NDMM patients is listed in **Table S1**. Informed consent was obtained from all study subjects themselves and voluntarily signed an informed consent form, the contents of which were reviewed and approved by the Ethics Committee of Tianjin Medical University General Hospital.

**2 Antibodies and reagents**

The antibodies and reagents used included: Flow cytometry antibodies, Western-blot antibodies, Tim-3 inhibitor, and Exosome inhibitor. All these antibodies and reagents were shown in **Table S9**.

**3 Cell culture**

**3.1 Isolation and culture of BMSCs**

First, bone marrow mononuclear cells (BMMNCs) from BM aspirations of NDMM patients and healthy controls were separated using density gradient centrifugation. The BMMNCs were counted, seeded in 6-well plates at a density of 1×10^6^ cells/cm2, and cultivated at 37 °C in humidified air containing 5% CO2. The BMMNCs were cultured in Dulbecco's modified Eagle's medium/F12 medium (GIBCO, Darmstadt, Germany) supplemented with 20% fetal bovine serum (FBS), 100 g/ml penicillin, and 100 U/ml streptomycin (GIBCO). Then, the medium was changed every other day. When the connected cells achieved 90% confluence, BMSCs were released using trypsin-ethylenediamine tetraacetic acid (EDTA) (GIBCO) and then cultured to three passages.

**3.2 Culture bone marrow NK cells**

NK cells were isolated from bone marrow of NDMM patients. Up to 5 mL of EDTA bone marrow from NDMM patients was collected and mononuclear cells were isolated by lymphocyte separation liquid. Samples were then washed twice, per 1×10^8^cells, and then resuspended in 300μL running buffer. NK cells were purified from mononuclear cells using a NK cell Isolation Kit (Miltenyi Biotec, Germany) according to the manufacturer's instructions. Analyzing the purity of bone marrow NK cells by FCM. NK cells were cultured in a humidified atmosphere (37.5 ℃ and 5% CO2), using RPMI 1640 medium (Boehringer, Ingelheim, Germany) containing 20% fetal calf serum (FBS, Gibco, California, USA), 100ug/mL penicillin (Gibco), 100U/mL streptomycin (Gibco) and 200 U/ml IL-2.

**3.3 Culture MM U266 and RPMI-8226 cells**

MM U266 and RPMI-8226 cells were purchased from the Tumor Cell Bank of the Chinese Academy of Medical Sciences (Beijing, China) and were cultured in RPMI‐1640 media (GIBCO) supplemented with 15% FBS (GIBCO), 100 g/ml penicillin (GIBCO) and 100 U/ml streptomycin (GIBCO) in humidified air with 5% CO2 at 37˚C. The media were replaced every 2–3 days.

**3.4 Direct co-culture of bone marrow NK cells with BMSCs**

BMSCs were cultured to third-generation maturity, while bone marrow NK cells were cultured to availability. Co-culture system of BMSCs/NK cells were constructed.

BMSCs/NK direct co-culture: group A: NK alone; group B: NK+F38-2E2; group C: NK+BMSCs; group D: NK+BMSCs+F38-2E2. BMSCs and NK cells at 1:4 ratio. NK cell functions in each group were detected by flow-cytometry (FCM) after co-culture for 6d.

**3.5 Indirect co-culture of bone marrow NK cells with BMSCs**

BMSCs were cultured to third-generation maturity, while bone marrow NK cells were cultured to availability. Co-culture system of BMSCs/NK cells were constructed.

BMSCs/NK indirect co-culture: group a: NK+U266 in small chamber, none in lower chamber; group b: NK+U266 in small chamber, BMSCs in lower chamber; group c: NK+U266+F38-2E2 in small chamber, BMSCs in lower chamber; group d: NK+U266 in small chamber, BMSCs+GW4869 in lower chamber. BMSCs and NK cells at 1:4 ratio. NK cell functions in each group were detected by FCM after co-culture for 6d.

**3.6 Co-culture of bone marrow NK cells with galectin-9 knockdown BMSCs, BMSCs-derived exosomes and galectin-9 knockdown BMSCs-derived exosomes**

Group I: NK alone; group II: NK+BMSCs; group III: NK+galectin-9 knockdown BMSCs; group IV: NK+BMSCs-derived exosomes; group V: NK+galectin-9 knockdown BMSCs-derived exosomes. Add 100ug protein number of exosomes per 1ml of culture medium.

**3.7 Co-culture of bone marrow NK cells with U266 cells**

MM U266 cells were added to the above co-culture systems. U266 cells and bone marrow NK cells/NK-92 cells at 1:1 ratio. Apoptosis ratios of U266 cells in each group were detected by flow-cytometry after co-culture for 72h.

**4 Flow-cytometry**

NK cell quantity and Tim-3 expression: Fresh bone marrow was collected and supernatant was removed by centrifugation, erythrocytes were lysed, 5 μL of CD3-PerCp, CD56-PE, and CD16-FITC antibodies were added, incubated for 15 min at room temperature (RT) in dark to label NK cells, then 5 μL of Tim-3-APC antibody was added, incubated for 15 min at RT in dark, washed twice and then detected by FCM.

NK cell function: The expression of CD107a and NKG2D on the surface of NK cells and the expression of INF-γ and Perforin in NK cells were detected by FCM to evaluate the function of NK cells. After immunolabeling NK cells with CD3, CD56 and CD16, 5 μL of CD107a-PB450 and NKG2D-PC7 were added, incubated for 20 min, and the cells were washed twice and then detected by FCM. 12 hours before the detection of INF-γ and Perforin, 2 μL of cell stimulation cocktail (eBioscience, CatLog Number: 00-4975) was added to the samples with immunolabeled NK cells, and the cells were permeabilized with IntraSure kit (BD Biosciences). Then the cells were incubated with 5 μL of INF-γ-PC7 and Perforin-PB450 for 30 min, respectively. Finally, the cells were washed twice with PBS and detected with FCM.

U266 apoptosis assay: A FITC annexin V Apoptosis Detection Kit I (BD Biosciences) was used to detect the apoptosis of MM U266 cells. First, we harvested MM U266 cells from the co‐culture system. Then, the cells were washed with PBS twice and then resuspended with 1 × binding buffer at a concentration of 1× 10^6^ cells/ml for 30 min in the dark. Then, 5 µl of Annexin V-FITC and 5 µl of PI-PE were added and incubated for 10 min. Finally, the apoptosis of MM U266 cells was detected by FCM.

**5 Exosome isolation**

After the BMSCs were cultured to the 3rd generation, the medium was removed and replaced with medium containing exosome removal fetal bovine serum, and the medium was collected after about 3 d. The exosome removal fetal bovine serum was acquired by centrifuging at 120000×*g* at 4℃ for 18 hours via Ultracentrifuge (Optima XE, Beckman Coulter, USA).

The cell debris was removed by centrifugation at 1500 g for 15 min, the precipitates were collected by centrifugation at 100000 g for 70 min using an ultracentrifuge (Optima XE, Beckman, USA) and resuspended in 200 μL PBS. The exosome precipitates were isolated by a serum/plasma exosome kit (*TransExo*^TM^ Serum/Plasma Exosome Kit) according to the reagent manufacturer's instructions. Exosome precipitates were resuspended with 100 μL of PBS and stored at -80°C.

**6 Transmission electron microscope (TEM) analysis**

TEM was used to analysis the BMSCs-derived exosomes. Firstly, the exosomes were isolated by centrifugation. Secondly, the collected exosomes were stained, and then observed and captain images using a TEM (JEM-1200EX).

**7 siRNA knockdown of galectin-9 in BMSCs**

The three human galectin-9 siRNA were synthesized by GenePharma Company (Suchow, China). Sequence information is shown in the table S10. Add 50μL of serum-free medium to a 1.5ml sterile centrifuge tube, add 2μL of transfection reagent, mix well and leave for 5min; add 50μL of serum-free medium to another 1.5ml sterile centrifuge tube and add 20pmol galectin-9-RNA oligo, mix well and leave for 5min; add the transfection solution mixture to the RNA oligo mixture The transfection solution mixture was added to the RNA oligo mixture, left for 15min, and BMSCs were immediately transfected, and protein expression was detected at 48h.

**8 Western blot**

Western blot analysis was performed to evaluate the Galectine-9 protein concentrations in the BMSCs-derived exosomes. The exosomes were lysed on ice with lysate for exosome protein (NoninBio, Shanghai, China). Then, protein concentrations were determined via a bicinchoninic acid (BCA) protein assay kit (Dingguo Changsheng, Beijing, China). The proteins (30ug/lane) were separated using an 8% gels SDS–polyacrylamide gel electrophoresis (SDS-PAGE). The separated proteins were transferred to a polyvinylidene difluoride (PVDF) membrane (Solarbio, Beijing, China). PVDF membranes were blocked with a solution containing 5% skim milk and incubated overnight at 4 °C using the following antibodies: CD63 (NoninBio, Shanghai, China), TSG101 (NoninBio, Shanghai, China), Galectine-9 (Abcam, USA) and β-Tubulin (Cell Signaling Technology, USA). Above antibodies were diluted at 1:1000 in 5% bovine serum albumin (BSA). The PVDF membranes were washed three times with Tris-buffered saline [0.1% Tween-20 (TBST, Solarbio, Beijing, China)], incubated for 1 h at RT with horseradish peroxidase conjugated anti-rabbit IgG sheep antibody (1:5000 dilution in 5% BSA; Cell Signaling Technology, Danvers, MA, USA), and washed another three times with TBST. The protein bands were visualized using a chemiluminescence kit (Solarbio, Beijing, China).

**9 Statistical analyses**

The independent sample t-test was used for two groups of mutually independent random samples that conform to normal distribution, one-way analysis of variance (ANOVA test) is used for more than two groups of mutually independent random samples that obey normal distribution, and the paired t-test is used for data of paired samples. Pearson correlation analysis was used for correlations between different indicators of the two groups. All statistical analyses were done with SPSS software for Windows (version 24.0), and all statistical plots were done with GraphPad Prism software for Windows (version 8.0). Statistical significance: *p* value < 0.05 indicates statistical significance.
